# BRB-seq: ultra-affordable high-throughput transcriptomics enabled by bulk RNA barcoding and sequencing

**DOI:** 10.1186/s13059-019-1671-x

**Published:** 2019-04-19

**Authors:** Daniel Alpern, Vincent Gardeux, Julie Russeil, Bastien Mangeat, Antonio C. A. Meireles-Filho, Romane Breysse, David Hacker, Bart Deplancke

**Affiliations:** 1 Institute of Bioengineering, School of Life Sciences, École Polytechnique Fédérale de Lausanne (EPFL), CH-1015 Lausanne, Switzerland; 2 Swiss Institute of Bioinformatics, CH-1015 Lausanne, Switzerland; 3 Gene Expression Core Facility, School of Life Sciences, École Polytechnique Fédérale de Lausanne (EPFL), CH-1015 Lausanne, Switzerland; 4 Protein Expression Core Facility, School of Life Sciences, École Polytechnique Fédérale de Lausanne (EPFL), CH-1015 Lausanne, Switzerland

**Keywords:** RNA-seq, Transcriptomics, Gene expression, qPCR, Barcoding

## Abstract

Despite its widespread use, RNA-seq is still too laborious and expensive to replace RT-qPCR as the default gene expression analysis method. We present a novel approach, BRB-seq, which uses early multiplexing to produce 3′ cDNA libraries for dozens of samples, requiring just 2 hours of hands-on time. BRB-seq has a comparable performance to the standard TruSeq approach while showing greater tolerance for lower RNA quality and being up to 25 times cheaper. We anticipate that BRB-seq will transform basic laboratory practice given its capacity to generate genome-wide transcriptomic data at a similar cost as profiling four genes using RT-qPCR.

**Electronic supplementary material:**

The online version of this article (10.1186/s13059-019-1671-x) contains supplementary material, which is available to authorized users.

## Background

High-throughput sequencing has become the method of choice for genome-wide transcriptomic analyses as its price has substantially decreased over the last years. Nevertheless, the high cost of standard RNA library preparation and the complexity of the underlying data analysis still prevent this approach from becoming as routine as quantitative (q) PCR, especially when many samples need to be analyzed. To alleviate this high cost, the emerging single-cell transcriptomics field implemented the sample barcoding/early multiplexing principle. This reduces both the RNA-seq cost and preparation time by allowing the generation of a single sequencing library that contains multiple distinct samples/cells [1]. Such a strategy could also be of value to reduce the cost and processing time of bulk RNA sequencing of large sets of samples [2–5]. However, there have been surprisingly few efforts to explicitly adapt and validate the early-stage multiplexing protocols for reliable and cheap profiling of bulk RNA samples.

All RNA-seq library preparation methods are globally relying on the same molecular steps, such as reverse transcription (RT), fragmentation, indexing, and amplification. However, when compared side by side, one can observe variation in the order and refinement of these steps (Additional file 1: Figure S1a). Currently, the de facto standard workflow for bulk transcriptomics is the directional dUTP approach [6, 7] and its commercial adaptation “Illumina TruSeq Stranded mRNA”. Both procedures evoke late multiplexing, which necessitates the processing of samples on a one-by-one basis. To overcome this limitation, the RNAtag-seq protocol implemented the barcoding of fragmented RNA samples, which allows for early multiplexing and generation of a sequencing library covering entire transcripts [8]. However, this protocol involves rRNA-depletion and bias-prone RNA adapter ligation [9], which is relatively cumbersome and expensive. Although providing a significantly faster and cheaper alternative, other approaches such as QuantSeq (Lexogen) and LM-seq still require the user to handle every sample individually [10] (Additional file 1: Figure S1a).

In contrast, early multiplexing protocols designed for single-cell RNA profiling (CEL-seq2, SCRB-seq, and STRT-seq) provide a great capacity for transforming large sets of samples into a unique sequencing library [11–13]. This is achieved by introducing a sample-specific barcode during the RT reaction using a 6–8 nt tag carried by either the oligo-dT or the template switch oligo (TSO). After individual samples have been labeled, they are pooled together, and the remaining steps are performed in bulk, thus shortening the time and cost of library preparation. Since the label is introduced to the terminal part of the transcript prior to fragmentation, the reads solely cover the 3′ or 5′ end of the transcripts. Therefore, the principal limitation of this group of methods is the incapacity to address splicing, fusion genes, or RNA editing-related research questions. However, most transcriptomics studies do not require or exploit full transcript information, implying that standard RNA-seq methods tend to generate more information than is typically required. This unnecessarily inflates the overall experimental cost, rationalizing why 3′-end profiling approaches such as the 3′ digital gene expression (3′DGE) assay have already been proven effective to determine genome-wide gene expression levels, although with a slightly lower sensitivity than conventional mRNA-seq [14].

In this study, we set out to generate a method for affordable, efficient, and accurate bulk RNA profiling of a large number of samples that combines the high-throughput capacity of single-cell transcriptomics and the high performance of standard RNA-seq. As our experimental foundation, we selected the SCRB-seq approach [13], a single-cell transcriptomics protocol that we deemed to be the most time- and cost-effective amongst all early multiplexing approaches (Additional file 1: Figure S1a,b). Moreover, its unaltered workflow had already been used in several studies for bulk RNA profiling [14–20]. Our own benchmarking efforts of bulk SCRB-seq revealed however important quality issues, prompting us to test and improve key steps of this workflow (Additional file 1: Figure S1b), including the barcoded primer design, initial RNA amount, number of amplification cycles, and tagmentation strategies, culminating into the presented Bulk RNA Barcoding and sequencing (BRB-seq) approach. We further assessed the performance of BRB-seq relative to Illumina TruSeq, the standard for analyzing bulk RNA samples, and found that BRB-seq is highly reliable for all assessed quality markers and displays high performance, even on fragmented RNA samples.

## Results

### Adaptation of the early multiplexing RNA-seq library preparation workflow

First, we set out to benchmark SCRB-seq against the “gold standard” Illumina TruSeq workflow for bulk gene expression profiling. To do so, we prepared libraries following both protocols using RNA from GM12878 cells treated with either DMSO or IKK inhibitor (BAY 11-7082) to induce gene expression differences and thus to assess a potential difference between these two methods in the power to detect differentially expressed genes starting from the same RNA.

**Fig. 1:**
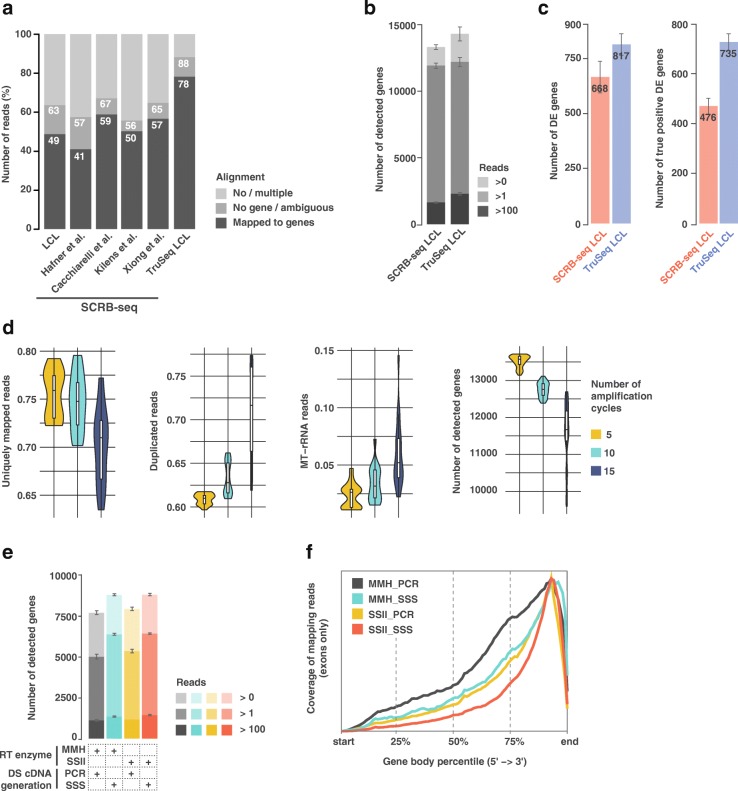
Global assessment of SCRB-seq’s performance for bulk RNA-seq. **a** Comparison of read alignment performances between TruSeq and five SCRB-seq datasets: one lymphoblastoid cell line (LCL; generated in-house), and four public datasets from [15, 18]. The no/multiple alignment values are derived from the STAR [35] alignment, and no gene/ambiguous and mapped to genes correspond to the annotation of the reads to the genes by Htseq [49]. **b** Total number of detected genes in the same LCL RNA samples by SCRB-seq and TruSeq at different detection thresholds (e.g., “Reads > 0” means that a gene is considered detected if it is covered by at least one read). **c** Evaluation of SCRB-seq’s performance relative to TruSeq using the data downsampled to 1M single-end reads and shown by the total number of identified DE genes and number of “true positive” DE genes. The latter represents a subset of DE genes identified using the full TruSeq 30M paired-end set; the error bars correspond to the variation produced by downsampled replicates (see the “Methods” section). **d** Assessment of the impact of the number of cycles during PCR pre-amplification of SCRB-seq libraries (downsampled to 1M single-end reads) prepared with BU3 primers. Performances were evaluated through variable quality measures: uniquely mapped reads, level of duplication, rate of MT-rRNA reads, and number of detected genes. **e** Assessment of the complexity of the libraries (downsampled to 100k single-end reads) obtained with different combinations of RT enzymes and DS cDNA generation procedures at various detection cutoffs (e.g., “Reads > 0” means that a gene is considered detected if it is covered by at least one read). **f** Read coverage across the gene body for different combinations of RT enzymes and DS cDNA generation procedures. Legend: DS cDNA, double-stranded cDNA; SE, single end; MMH, Maxima Fermentas Minus H Enzyme; SSII, Superscript II enzyme; SSS, second-strand synthesis using Nick translation; PCR, pre-amplification by polymerase chain reaction

After sequencing, we first observed approximately 30% less SCRB-seq reads mapping to genes as compared to TruSeq (Fig. 1a), which implies that SCRB-seq libraries are more “contaminated” with undesired sequences (such as oligos, adapters, or polyA). This leads to a loss of approximately half of the initial sequenced reads, which may unnecessarily increase the sequencing need and thus overall cost. Interestingly, this effect was reproduced when aligning four publicly available bulk SCRB-seq datasets [14–16, 18] (Fig. 1a and Additional file 2: Table S1). Subsequently, we downsampled the respective libraries *after alignment* to consider an equal number of reads per replicate for both libraries (1M aligned reads, see the “Methods” section) and thus to allow a fair comparison between the SCRB-seq and TruSeq methods, thereby correcting for the discussed alignment issues. Upon investigating the complexity of the libraries (i.e., the number of detected genes), we found that at similar read depth (1M reads), SCRB-seq detected significantly less expressed genes than TruSeq (7% less genes across two conditions and three replicates, *t* test *p* value = 0.0038), thus revealing lower library complexity (Fig. 1b). We then performed an empirical power analysis between the two conditions of our LCL experiment (DMSO- or BAY 11-7082-treated LCL cells). We found that, with the same processed RNA, the SCRB-seq protocol uncovered ~ 20% less total differential expressed (DE) genes than the 1M downsampled TruSeq (Fig. 1c, 10 random downsampling). More importantly, the downsampled TruSeq was able to uncover ~ 35% more DE genes that were deemed “true positives” because these were uncovered using the full collection of 30M paired-end TruSeq reads. This points to a lower sensitivity of SCRB-seq libraries (less true positives/more false negatives). We concluded that in its original form, SCRB-seq is not competitive with TruSeq and that important workflow adaptations would be required to use this approach for bulk RNA sequencing.

Notably, we also noticed increased occurrences of “T” bases in the UMI sequence in the proximity of the dT stretch (Additional file 1: Figure S1c, left and center panels). We reasoned that since the stretch of 30 dT was not separated from the UMI sequence in the E3V6NEXT oligo-dT primer, oligonucleotides with longer dT had a higher affinity to the poly-A RNA tail, thus potentially affecting the diversity of the reads. This caused enhanced incorporation of primers containing UMIs and barcodes with higher dT, biasing the data. To overcome this issue, we designed novel BU3 primers so that the UMI and oligo-dT sequences were separated by five random non-T nucleotides (“V”), thus increasing the total UMI length to 15 nt (10 “N” + 5 “V”). This proved to be sufficient to reduce the overrepresentation of “T”-containing UMIs (Additional file 1: Figure S1c, right panel).

In addition, we anticipated that the efficiency of tagmentation might be increased by using Tn5 enzyme loaded with only i5 compatible adapters. Nextera Tn5 is a mix of transposases with two different adapter sequences (Tn5-A/B) intended to append either i5 or i7 Illumina indexes to generate compatible sequencing libraries. However, since the SCRB-seq libraries are amplified using only the i7 adapter (and a custom P5-TSO, bearing a P5 capture sequence), the cDNA fragments produced by introduction of the i5 compatible adapter sequence by Tn5 complex are not amplified by the limited-cycle PCR due to suppression PCR and are thus lost [21]. To reduce this loss, we used Tn5 enzymes that were produced in-house following the protocol of [22]. Indeed, we observed an increased library yield when in-house Tn5-B/B (loaded with only i7 compatible adapters) was used, compared to either Tn5 bearing both adapters, in-house made Tn5-A/B or the Nextera (Additional file 1: Figure S1d). Therefore, the use of in-house produced Tn5 helped to reduce the cost of library preparations. However, the impact of the Tn5 enzyme (A/B or B/B) on the sequencing data quality appeared to be relatively minor as confirmed by the downstream analysis (Additional file 1: Figure S2d), implying that one could still use Nextera Tn5 enzyme without loss of quality of the final data.

### Second-strand synthesis without amplification improves data quality and biological relevance

Next, we performed a systematic evaluation of the key steps that might potentially affect the performance of SCRB-seq (Additional file 1: Figure S1b). To do so, we turned to a familiar model system that was also used in the original SCRB-seq paper [13]: adipocyte formation from human adipose stromal cells (hASCs), since a large number of genes show differential expression along this differentiation trajectory [23]. Specifically, we isolated total RNA from hASCs at two adipogenesis time points: t0 and t14 (non-differentiated ASCs and adipocytes, respectively) with two technical replicates each (Additional file 1: Figure S2a) after which we prepared cDNA libraries using our own set of improved barcoded primers (BU3).

We first tested different pre-amplification PCR cycle numbers (5, 10, and 15) as well as different input RNA amounts (1, 10, 100, 500, 1000, and 2000 ng), which may affect the overall amplification efficiency (Fig. 1d and Additional file 1: Figure S2b). To test the required combination of conditions, we prepared 18 libraries involving altogether 72 samples. This yielded two important insights: first, we detected an inverse correlation between the complexity/diversity of our RNA-seq libraries and the number of PCR cycles that were used to generate full-length double-stranded cDNA (Fig. 1d). Second, this effect was essentially independent of RNA input amount, although the highest performance in terms of uniquely mapped reads, percent duplication, mitochondrial read contamination, and the number of detected genes was generally observed between 10 and 100 ng of input RNA (Additional file 1: Figure S2b). Thus, five amplification cycles using 10–100 ng of input RNA appears preferred. We further found that this conclusion is independent of the RT enzyme used, since replacing Maxima Minus H (MMH) with SuperScript II (SSII) did not alter the number of detected genes using five amplification cycles and 100 ng of input RNA (Fig. 1e). Finally, our data revealed that the post-tagmentation library amplification step has a relatively minor impact on the downstream quality of the results as exemplified by solely 1–2% variation in read alignment rate and number of identified genes across the libraries amplified 8 to 12 PCR cycles (Additional file 1: Figure S2c).

**Fig. 2:**
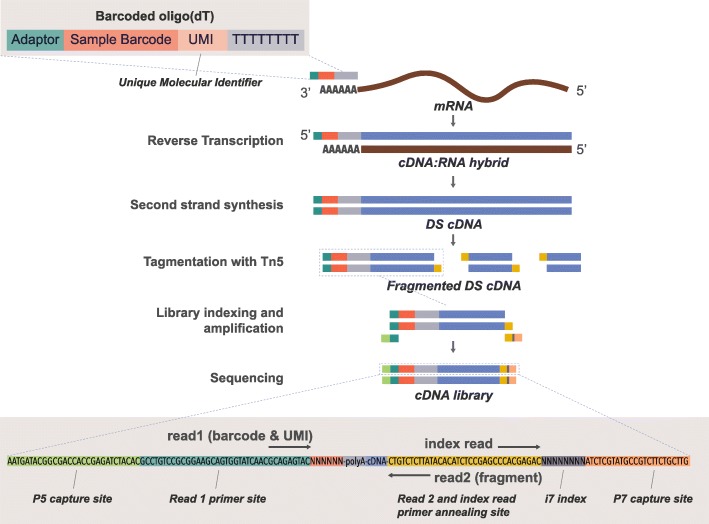
Schematic overview of the BRB-seq protocol. This schema highlights in details all steps of the final BRB-seq protocol. The bottom grayed window shows the final BRB-seq construct used for Illumina sequencing. The read Read1 and Read2 primers are used to sequence the barcode/UMI and cDNA fragment respectively. Index read (i7) is used to demultiplex Illumina libraries. Legend: DS cDNA, double-stranded cDNA

The lowering data quality upon increasing the number of amplification cycles made us wonder whether PCR amplification in general is decreasing the quality of the output data. We therefore explored the value of using the Gubler-Hoffman procedure [24] to generate double-stranded cDNA instead of PCR amplification. While PCR amplification is easier to implement, the Gubler-Hoffman method bypasses the need for including a template switch oligo (TSO) in the first-strand synthesis, since the second-strand generation is driven by RNA primer-dependent nick translation by DNA polymerase I. Moreover, since we work with bulk RNA, samples may not require substantial amplification to enable subsequent tagmentation. In addition, for the remainder of the experiments, we used 100 ng of input RNA given the results discussed above and given that such an amount appears compatible with the majority of bulk RNA sequencing projects. As expected, we found that the yield of full-length cDNA generated with nick translation is lower compared to that obtained with PCR amplification and is dependent on the RT enzyme used (MMH or SSII) (Additional file 1: Figure S3a). Moreover, libraries that were generated with nick translation were more concentrated at the 3′-end of transcripts, an effect that was most visible when using SSII (Fig. 1f). The latter enzyme also yielded a lower rate of MT-rRNA reads compared to MMH (Additional file 1: Figure S3b). This is in line with the previously reported higher enzymatic activity of MMH compared to SSII [25], which may explain its lower specificity. Moreover, libraries prepared with nick translation involving the SSII enzyme had an increased ratio of reads mapping to annotated genes, namely ~ 76%, compared to ~ 65–70% produced with PCR amplification or when using the MMH enzyme (Additional file 1: Figure S3c). This was caused by a lower bias/noise resulting from the lower adapter and polyA contamination when preparing libraries using nick translation compared to pre-amplification (Additional file 1: Figure S3d). We concluded that second-strand synthesis via nick translation with SSII is preferable over the other combinations of second-strand synthesis/enzymes. These observations rationalize the novel Bulk RNA Barcoding and sequencing (BRB-seq) workflow, which features modified oligo-dT for cDNA barcoding and the second-strand synthesis involving DNA PolI Nick translation instead of PCR which accordingly enables the elimination of TSO for the first-strand synthesis (Fig. 2). The sequencing library is then prepared using cDNA tagmented by an in-house B/B Tn5 transposase and further enriched by limited-cycle PCR with Illumina compatible adapters.

### BRB-seq outperforms SCRB-seq and its power is comparable to that of TruSeq

**Fig. 3:**
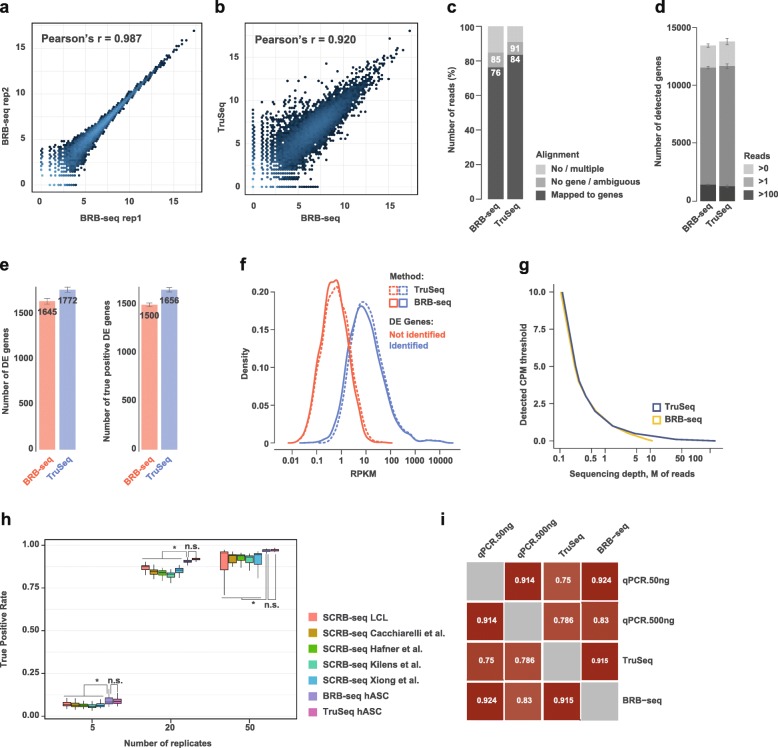
BRB-seq’s overall performance relative to TruSeq. **a** Correlation of log2 read counts between technical replicates at t14 for the BRB-seq workflow (Pearson correlation *r* = 0.987). **b** Correlation of log2 read counts between BRB-seq and TruSeq (Pearson correlation *r* = 0.920). **c** Comparison of read alignment performances between BRB-seq and TruSeq. The no/multiple alignment values are derived from the STAR [35] alignment, and no gene/ambiguous and mapped to genes correspond to the annotation of the reads to the genes by Htseq [49]. **d** Comparison of library complexity between BRB-seq and TruSeq (e.g., “Reads > 0” means that a gene is considered detected if it is covered by at least one read). **e** Evaluation of BRB-seq’s performance relative to TruSeq using the data downsampled to 1M single-end reads and shown by the total number of identified DE genes and the number of “true positive” DE genes. The latter represents a subset of DE genes identified using the full TruSeq 30M paired-end set (see the “Methods” section). **f** The distribution of RPKM levels of expression of the DE genes detected (blue) or not detected (red) in the downsampled TruSeq (dotted) or BRB-seq (plain) that overlaps with the “gold standard” TruSeq ~ 30M paired-end reads. **g** The sequencing depth required for detecting genes with a given CPM expression level using TruSeq and BRB-seq libraries. A sequencing depth is considered sufficient if the gene is detected more than 95% of the time. **h** Power simulation analysis of public and in-house bulk SCRB-seq, BRB-seq, and TruSeq datasets (*p < 0.001; n.s. non-significant). **i** Correlation of expression values (normalized to *HPRT1*) determined by qPCR (in replicates, with 50 ng and 500 ng of total RNA used per RT), TruSeq and BRB-seq. Pearson’s *r* values are indicated. In all panels, for an unbiased comparison, all libraries were randomly downsampled to one million single-end reads (see the “Methods” section)

Next, we aimed at benchmarking our newly developed BRB-seq approach by comparing its output data to a reference “gold standard” dataset. To do so, we used again the Illumina TruSeq Stranded mRNA protocol and applied it on the same hASC RNA samples (Additional file 1: Figure S2a). First, we observed a high correlation between log2 transformed read count values of technical BRB-seq replicates (Pearson’s *r* = 0.98) (Fig. 3a) and similarly with TruSeq (*r* = 0.92) (Fig. 3b). The ratio of reads mapping to annotated genes was slightly lower than that of TruSeq (~ 76% vs. ~ 84%, Fig. 3c), but on average 22% higher than what was previously observed when using the original SCRB-seq protocol (Fig. 1a). The BRB-seq libraries showed high read diversity, allowing the detection of a comparable number of genes as TruSeq at the same sequencing depth (Fig. 3d). Importantly, we confirmed the high accuracy of DE gene detection of BRB-seq validated by the high number of DE genes overlapping with TruSeq (Fig. 3e). The latter detected only 7% more DE genes than BRB-seq, compared to 35% more than SCRB-seq (Fig. 1c). BRB-seq’s efficacy was further confirmed by increased fold change (t0 vs t4) correlation, as well as PR AUC and ROC AUC values (Additional file 1: Figure S4a, taking the full TruSeq ~ 30M paired-end run as “gold standard”). Importantly, we found that the ability to detect DE genes is inherently linked to the absolute gene expression levels and both TruSeq and BRB-seq exhibited very similar detection thresholds (Fig. 3f). We, therefore, concluded that a greater sequencing depth (> 5M reads) would in this case only be effective for BRB-seq or TruSeq libraries when specifically looking for DE genes with low to very low expression levels (i.e., CPM < < 1) (Fig. 3g).

We further investigated whether DE genes that were discovered with the two approaches were biologically relevant. For this, we conducted a functional enrichment analysis of the DE genes that were upregulated in the differentiated hASC cells using adipocyte-related gene sets from KEGG [38], Gene Ontology (GO) [37], and Gene Atlas databases. Overall, both BRB-seq and TruSeq DE genes were strongly enriched in adipocyte gene sets (Additional file 1: Figure S4b). It is also worth noting that the “Adipocyte” gene set (from Gene Atlas database) was slightly more enriched with BRB-seq as compared to TruSeq at a similar sequencing depth.

After having empirically validated the capacity of BRB-seq on real data, we aimed at evaluating its ability to uncover DE genes based on simulated data, where the DE genes are a priori known. To this end, we performed a power simulation using the powsimR package [26]. We thereby included, for the sake of comprehensiveness, not only our in-house generated data (SCRB-seq LCL, BRB-seq hASC, and TruSeq hASC) but also the published SCRB-seq datasets mentioned above [14–16, 18] since the DE genes are simulated. We performed the simulation using 5, 20, and 50 replicates downsampled at 1M reads (see the “Methods” section). The results of this analysis proved concordant with our empirical power analysis, showing again that BRB-seq was able to uncover DE genes at a level comparable with TruSeq (*t* test *p* value n.s.), while significantly higher than that of SCRB-seq (*t* test *p* < 0.05 for all three studies), and the effect is maintained for different numbers of replicates (Fig. 3h).

Given the performance of BRB-seq, combined with the fact that it is time- and cost-efficient, we envisioned that it could potentially become an alternative to RT-qPCR assays, especially when large sets of samples need to be profiled. To confirm that BRB-seq libraries can produce reliable gene expression results, we compared it to RT-qPCR data. We evaluated nine genes that are expressed at different levels in adipocytes. We performed two RT-qPCR replicates, one with 50 ng of RNA and the other with 500 ng using again the same RNA sample as was used to prepare the first-strand reactions for BRB-seq and TruSeq libraries (Additional file 1: Figure S2a). After normalization to *HPRT1* expression, we assessed the correlation of expression values between each of the methods (Fig. 3i). We observed that both BRB-seq and TruSeq highly correlate with qPCR (Pearson’s *r* = 0.8–0.9) with BRB-seq slightly outperforming TruSeq. This effect was observed for both qPCR replicates.

Taken together, these results confirm the high overall performance of the BRB-seq approach, which yields a comparable efficiency/sensitivity as TruSeq, but at a fraction of its cost (see the “Discussion” section).

### Multiplexing capacity of BRB-seq

So far, our experiments involved just a couple of samples. To assess whether BRB-seq’s performance would be maintained in a multiplexing context, we prepared an additional BRB-seq library containing 60 human lymphoblastoid cell line (LCL) samples, which have been routinely used in large-scale projects including the 1000 Genome Project. We focused on these cell lines since corresponding Illumina TruSeq data had been generated at two separate occasions, thus enabling a direct, comprehensive comparison between the two approaches. Specifically, we used two datasets: “TruSeq A” is from [27] involving all 60 samples that were profiled with BRB-seq and “TruSeq B” from [28] containing 53 of the 60 samples (Additional file 2: Table S2). Of note, the libraries of both TruSeq datasets were prepared using TruSeq RNA Sample Prep Kit v2, which does not preserve strand-specific information, contrary to the BRB-seq and TruSeq mRNA Stranded protocols that were used before. However, given that only poly-A+ transcripts are profiled, we assume that differences in DE power between these TruSeq protocols are rather minor.

**Fig. 4:**
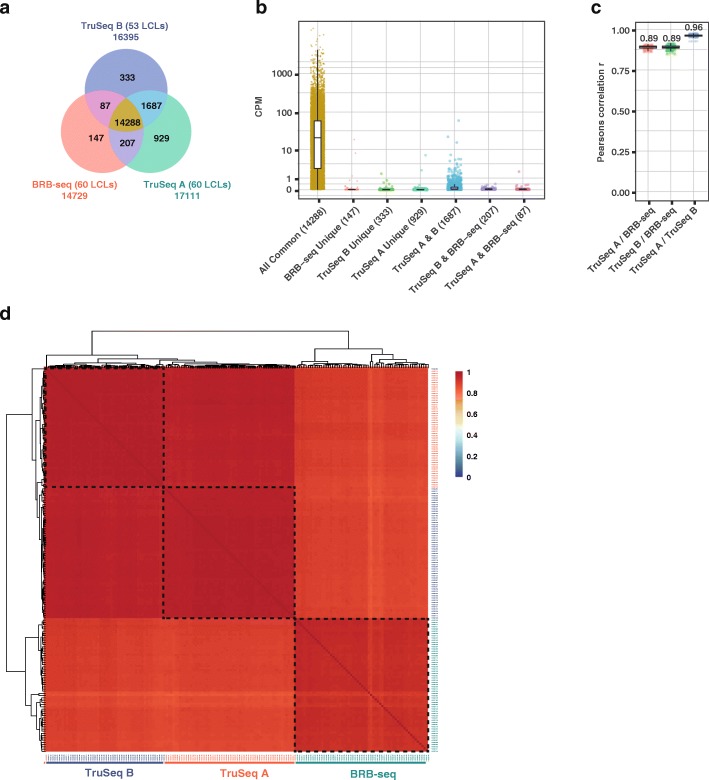
BRB-seq multiplexing experiment and comparison with TruSeq. **a** Venn diagram showing the protein-coding genes detected (at least one read) across all 60 (TruSeq A) or 53 (TruSeq B) LCL samples after downsampling to 1M reads. **b** Distribution of counts per millions (CPM) of genes taken from every subset (corresponding color) of the Venn diagram shown in panel **a**. **c** Pearson’s correlations of log2 expressions, calculated sample by sample, i.e., of the same sample taken from two different dataset combinations (TruSeq A and B and BRB-seq). **d** Correlation heatmap showing in greater detail the individual LCL sample correlations between all three datasets (BRB-seq, TruSeq A, and TruSeq B). Highlighted in black are the three main clusters, showing, as expected, a clear separation by protocol (BRB-seq vs. TruSeq) or sequencing run (TruSeq A vs. B), overriding the relatively modest biological differences between 60 LCL samples, while maintaining an overall high correlation (Pearson’s *r* > 0.8). In all panels, all libraries were randomly downsampled to one million single-end reads for an unbiased comparison (see the “Methods” section)

Our analyses showed that BRB-seq libraries identified over 14k protein-coding genes across the 60 samples (i.e., detected in at least one sample). The fraction of genes detected within all three datasets (Fig. 4a, yellow sector) represented over 97% of BRB-seq genes and 84–87% of the genes discovered by TruSeq. Importantly, this overlapping population contained *all* highly expressed genes (CPM > 100), *all but 54* medium-expressed genes (1 < CPM < 100, Fig. 4b, blue population), and *over 2600* lowly expressed genes (CPM < 1, Fig. 4b, yellow population). Thus, the genes that remained undetected by BRB-seq (1687 genes, Fig. 4a and Fig. 4b, blue population) contained predominantly lowly expressed genes (*n* = 1637, CPM < 1) and no highly expressed genes (CPM > 100). This likely reflects the fact that BRB-seq was initially sequenced to a lower level (6M single-end reads per sample on average) compared to TruSeq (13.6M and 29.7M paired-end reads for TruSeq A and B, respectively). Even prior to downsampling to 1M reads, therefore, some lowly expressed genes may not have been sequenced enough to aggregate at least one read in the BRB-seq dataset and thus may also not be detectable upon downsampling. Similarly, most genes that were uniquely identified within each dataset, including by BRB-seq, tend to be lowly expressed (CPM < 1) (Fig. 4b).

We further found an overall high correlation between BRB-seq and TruSeq A and B log2 read count values (Pearson’s *r* = 0.89 and 0.89, Fig. 4c), performed for each replicate sample across protocols. Finally, across the samples, the overall correlation was above 0.8 and only slightly lower compared to what was found for the two TruSeq datasets (Fig. 4d).

Taken together, these results show that BRB-seq constitutes a highly affordable (see the “Discussion” section), robust high-throughput 3′-end transcriptomics approach that produces data featuring a quality that is comparable to that of the “gold standard” TruSeq methods.

### BRB-seq performs well on low-quality RNA samples

It is well established that the TruSeq Stranded mRNA method performs poorly on degraded RNA samples given the intrinsic requirement of this method to have an RNA quality number (equal to RIN, RNA integrity number) ≥ 7–8. This may reflect the fact that full-length transcripts are sequenced, thus requiring high-quality, intact RNA for accurate detection and quantification. Since 3′ RNA fragment quantification is known to be a robust way to estimate differential gene expression in samples with low RNA quality numbers (RQNs) [29], we decided to evaluate the performance of BRB-seq on fragmented RNA samples with low RQN values. For this, we employed chemical RNA fragmentation by incubation at 65 °C in the presence of Mg^++^ cations for 1 or 2 min, which resulted in a significant reduction in overall RNA size and RQN values (Additional file 1: Figure S5).

**Fig. 5:**
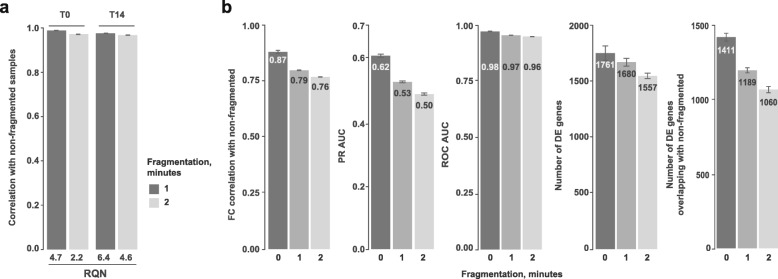
BRB-seq performance with fragmented RNA samples. **a** Pearson correlation between log2 read counts of intact (RNA quality number (RQN) = 8.9 and 9.8 for T0 and T14 respectively) versus fragmented samples (after 1 or 2 min of fragmentation). **b** Quality evaluation of BRB-seq libraries prepared with fragmented RNA samples (1 or 2 min fragmentation) compared with the intact RNA counterparts. For the analysis, the libraries were downsampled to 1M single-end reads (see the “Methods” section). “Max” threshold thus comes from the 1M downsampled intact RNA sample when compared to itself, without downsampling. Legend: RQN, RNA quality number (maximum is 10)

As expected, we observed a clear inverse correlation between the quality of the samples and their RQN values, but of minor effect size. Indeed, the correlation between fragmented and non-fragmented samples remained above 97%, even for samples with very low RQN (Fig. 5a). Detection of DE genes in the degraded versus intact samples was more substantially affected by prolonged fragmentation and observed by lowered fold change correlation, PR AUC, and number of detected DE genes (Fig. 5b). Nevertheless, we could still detect more than 75% of true DE genes in the samples with RQN values as low as 2.2, which is generally considered as a mark of very highly degraded RNA (Fig. 5b). Together, these data show that BRB-seq allows reliable differential gene expression and functional enrichment analyses, even on low-quality/degraded RNA samples.

### BRB-seq data analysis pipeline and considerations

**Fig. 6:**
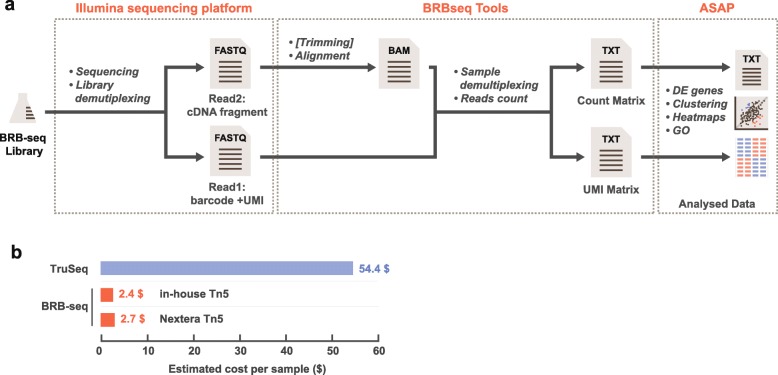
The streamlined BRB-seq data analysis workflow and its low cost. **a** Schematic representation of the BRB-seq library post-sequencing data processing pipeline. It includes the BRB-seqTools module (available on github, see the “Methods” section) that can perform optional read trimming, alignment, sample demultiplexing, and generation of a count table. The count table can be further analyzed by standard algorithms or loaded into ASAP, a web-based analytical interface that facilitates data exploration and visualization. **b** The estimated per sample cost of library preparation for 96 samples for TruSeq and BRB-seq. Per sample cost of BRB-seq involving in-house made Tn5 or Nextera Tn5 is indicated

Upon the sequencing of the BRB-seq libraries, highly multiplexed datasets are produced which may pose analytical problems, specifically for users with limited bioinformatic skills. To make the entire workflow of the method accessible to the scientific community at large, we aimed at streamlining the analysis of the sequenced data. For this, we developed a complete tool suite (http://github.com/DeplanckeLab/BRB-seqTools), supporting all the required post-sequencing tasks up until the generation of the read/UMI count matrix (Fig. 6a and detailed in Additional file 3: Supp. Method).

Thereafter, the data can be processed with conventional R scripts/packages to perform the required analyses or even Excel for direct visualization. Alternatively, the count matrix file can be supplied to ASAP (https://asap.epfl.ch/), a web-based platform devoted to comprehensive/automated transcriptome analyses developed in our lab [30]. Consequently, along with the protocol itself, we provide a seamless pre- and post-treatment pipeline for enabling any user to perform a state-of-the-art analysis of their BRB-seq data.

## Discussion

Building on experimental advances enabling single-cell gene expression profiling, we developed and validated a novel workflow to perform highly multiplexed Bulk RNA Barcoding and sequencing (BRB-seq). This required a series of optimizations from the original SCRB-seq workflow, which individually may be perceived as incremental, but which together culminated into a robustly benchmarked, large-scale bulk transcriptomics approach that produces data of superior quality than that provided by SCRB-seq. These adaptations include the elimination of template switch during the first-strand synthesis, allowing to alleviate the associated bias towards fully reverse transcribed molecules and potential artifacts related to strand invasion [31, 32]. Furthermore, we improved the design of barcoded oligo-dT primers and substituted the PCR amplification with the second-strand synthesis step. We demonstrate that these modifications substantially increased the complexity of the sequencing libraries, rendering the BRB-seq approach highly suitable for large-scale DE gene analysis, comparable to TruSeq’s DE gene detection performance, and with limited impact on its overall cost and hands-on time requirements.

We, therefore, think that BRB-seq promises to fill a so far unmet need for affordable transcriptomics of a large number of RNA samples. Indeed, our approach enables genome-wide gene expression analyses of dozens of samples simultaneously, in an effort- and cost-efficient manner. In our experience, up to 192 BRB-seq samples can be prepared by a single person within a day, given that the projected hands-on time is around 2 h. The number of samples in one library is scalable and merely depends on the number of available barcodes and desired sequencing depth per sample. Along with being fast and easily manageable, the protocol’s high advantage is its low cost of per sample library preparation, i.e., down to $2/sample if 96 samples are processed together (Fig. 6b and Additional file 2: Table S3). Adding the sequencing cost, we estimate the total expense to be around $20/transcriptome. This estimation is entirely guided by the type of desired analysis or organism and by the relative expression of specific genes of interest, which leaves sufficient space for optimization of sequencing depth and hence even greater cost reduction.

Importantly, the lower per sample cost of BRB-seq has several practical implications, such as the ability to (i) augment the experimental resolution by including more sampling points, (ii) validate gene expression dynamics on a genome-wide rather than on a per gene (qPCR) basis, and equally important, (iii) increase the number of experimental replicates. Indeed, at a fixed experimental cost, at least 10–20 times more samples can be considered when using BRB-seq compared to TruSeq. As shown by our power simulation analysis, the use of 20 instead of five replicates dramatically increases the power to detect DE genes (Fig. 3h). While biological or technical factors related to cell type, nature of replicates, RNA extraction procedure, sequencing, etc. may all influence the downstream results of such simulation analysis [33], the resulting data nevertheless revealed that BRB-seq’s performance to detect DE genes is greater than that of SCRB-seq and at par with that of TruSeq, thus favoring BRB-seq on a cost per sample basis. Of course, the actual number of experimental replicates will also depend on other factors including sample availability, but we anticipate that the cost for library preparation and sequencing will no longer present an important obstacle when designing a gene expression profiling experiment.

To implement BRB-seq, we recommend the total RNA content in the library pool to be in the range of 1–2 μg to reduce any potential performance variation of the second-strand synthesis step. This corresponds roughly to 10–20 ng per sample for a library of 96 samples (or 50–100 ng for 20). Our data also suggest that an input RNA amount as low as 1 ng might still produce a reliable library. However, we recommend in this case to pool multiple samples to assure that the cDNA is of sufficient quantity for tagmentation. As it is sometimes complicated to assess how deep a sample should be sequenced, we also provide an estimation of the number of sequencing reads that are required to detect a particular gene (95% chance of having at least 1 read), given its CPM expression (Fig. 3g).

The principal limitation of BRB-seq is the requirement to accurately assess RNA sample amounts prior to RT as any inter-sample variation will result in uneven distribution of sequencing reads. In our experience, this issue is solved through re-quantification of intermediate RNA dilutions that are prepared to normalize concentration variations.

Finally, BRB-seq features the UMI concept, which still needs to be further tested in the context of bulk experiments but can in principle increase the sensitivity of the results. This is in line with similar conclusions stating that the removal of UMI identical reads improves the FDR [20]. In particular, it can be used to overcome the amplification bias when samples with low RNA quantities (< 1 ng) need to be processed. Also, the UMI provides a good way of unbiased estimation of the duplication ratio, which is otherwise inflated with increasing sequencing depth (e.g., using Picard http://broadinstitute.github.io/picard/). It is worth noting that the user can modify the oligo and remove the UMI construct, or keep it but not sequence it for lowering costs.

## Conclusions

We anticipate that BRB-seq will become an attractive alternative for routine gene expression analysis and ultimately replace large RT-qPCR assays. Assuming that the current cost of one qPCR reaction is in the range of $1.5–2, the evaluation of the expression of three to four target genes in triplicate (~ 20 qPCR reactions) will cost approximately the same or even more than one full transcriptome analysis produced by BRB-seq, which involves library preparation and sequencing expenses. Importantly, low library preparation cost and time imply that more replicates can be profiled, which will greatly increase the statistical power underlying any DE analysis. Importantly, we provide simple data processing and analysis workflows that revoke the requirement for essential informatics skills. Once deployed, the setup can be used by experimental biologists to handle their data in a straightforward manner, therefore further streamlining the BRB-seq transcriptomics to the extent of a mere qPCR experiment.

## Methods

### Cell culture

The lymphoblastoid cell line GM12878 (Coriell Cat# GM12878, RRID:CVCL_7526) was cultured using RPMI 1640 medium supplemented with 10% fetal bovine serum and 1× penicillin/streptomycin. One million cells were treated with DMSO (Applichem #A3672,0250) or 3 μM BAY11-7082 (SignalChem, # C51-900) during 24 h prior to harvesting for RNA isolation.

hASCs were obtained from a fresh lipoaspirate as follows: 50 ml of lipoaspirate was washed twice with 40 ml of DPBS Ca+/Mg+ (Gibco, #14040091) in 100-ml syringes and incubated with 0.28 U/ml of liberase TM (Roche, #05401119001(ROC)) for 45 min at 37 °C under agitation. The digested tissue was mixed with 40 ml of CRB (1% human albumin (CSL Behring) in 40 ml of DPBS −/− (Gibco, #14190094)) and shaken vigorously to liberate the stromal cells. The aqueous phase was recovered and centrifuged at 400*g* for 5 min at RT. The cell pellet was resuspended in 15 ml of CRB and filtered through a 100-μm and then 40-μm cell strainer to ensure a single-cell preparation, centrifuged, and resuspended in Minimum Essential Medium (MEM) alpha (Gibco, #32561037) supplemented with 5% human platelet lysate (Cook Regentec, #G34936) and 50 μg/mL Primocin (InvivoGen, #ant-pm-1). hASCs were cultured in the same media composition until 70–80% confluency and detached using TrypLE Select (Life Technology, #1256311) for passaging.

For adipogenic differentiation, cells at confluence were treated with induction cocktail from Adipogenic BulletKit (Lonza, #PT-3004) for 7 days, followed by treatment with maintenance cocktail for another 7 days.

The LCL and hASC cultures were authenticated by microscopic morphology observation, and standard mycoplasma testing was performed using Hoechst dye fluorescent staining. The hASCs used for the experiment were passaged twice (P2) after isolation from the tissue sample.

### RNA samples for library preparation

Total RNA was isolated using TRI Reagent (Molecular Research Center, #TR118) followed by double precipitation with ethanol. The RNA concentration was determined using the Qubit RNA HS Assay Kit (Invitrogen, #Q32852), and integrity was assessed using a Fragment Analyzer (Advanced Analytical). The RNA from each differentiation time point was used in two technical replicates, resulting in four samples pooled per library. Libraries were prepared with the BRB-seq protocol using total RNA amounts ranging from 1 ng to 2 μg per sample (Additional file 2: Table S4).

RNA fragmentation was done using the NEBNext Magnesium RNA Fragmentation Module (NEB, #E6150S) with incubation time at 94 °C for 1 or 2 min. This resulted in RNA with a variable extent of degradation and corresponding RQN values.

A set of RNA samples from LCLs of the 1000 Genome Project was a generous gift from Manolis Dermitzakis (University of Geneva).

### RT-qPCR

For RT-qPCR, 50 ng or 500 ng of total RNA was used to generate the first strand using 1 μL of Superscript II (Invitrogen, #18064014) and 1 μL of anchored oligo-dT (ThermoFisher Scientific, #AB1247) in 20 μL total reaction mix following the protocol. cDNA was diluted five times using nuclease-free water, and 2 μL was used for each qPCR reaction. Quantitative real-time PCR was performed in three technical replicates on the ABI-7900HT Real-Time PCR System (Applied Biosystems) using the PowerUp SYBR Green Master Mix (Applied Biosystems, #A25742) using standard procedures. The qPCR primers for the target genes (*ADIPOQ*, *AXIN2*, *BCAT*, *CEBPB*, *FABP4*, *HPRT*, *LEP*, *LPL*, *PNPLA2*, and *PPARG*, see Additional file 2: Table S5) were designed with Primer3 software (RRID:SCR_003139) [34].

### BRB-seq protocol

#### First-strand synthesis

All the first-strand synthesis reactions were performed in 10 μL total volume using various amounts of RNA (50 pg–2 μg), 1 μL of 10 μM barcoded oligo-dT (BU3, Microsynth, for the list of oligos used see Additional file 2: Table S6 and S7), and either 0.125 μL of Maxima H Minus Reverse Transcriptase (MMH, ThermoFisher Scientific, #EP0753) or 0.25 μL Superscript II (SSII, Invitrogen, #180640). The reactions followed by the PCR pre-amplifications were complemented with 1 μL of 10 μM template switch oligo (TSO, IDT). RNA, BU3 primers, and 1 μL dNTP (0.2mM) were mixed together in a PCR plate, incubated at 65 °C for 5 min and then put on ice. The TSO, RT buffer (including 1 μL of DTT for the Superscript II protocol), and RT enzymes were added to each well, and the plates were incubated at 45 °C for 90 min for the Maxima protocol or 42 °C for 50 min followed by inactivation at 70 °C for 15 min for the Superscript II protocol. After RT, all the wells were pooled together and purified using the DNA Clean & Concentrator-5 kit (Zymo Research, #D4014) with 7× DNA binging buffer and single column. After elution with 20 μL of nuclease-free water, the samples were incubated with 1 μL Exonuclease I (NEB, #M0293) and 2 μL of 10× reaction buffer at 37 °C for 30 min, followed by enzyme inactivation at 80 °C for 20 min.

#### Second-strand synthesis

Double-stranded cDNA was generated by either PCR amplification (indicated as PCR in the text) or nick translation (indicated as SSS in the text) [24]. The PCR was performed in 50 μL total reaction volume using 20 μL of pooled and ExoI-treated first-strand reaction, 1 μL of 10 μM LA_oligo (Microsynth) primer, 1 μL of dNTP (0.2mM), 1 μL of with Advantage 2 Polymerase Mix (Clontech, #639206), 5 μL of Advantage 2 PCR buffer, and 22 μL of water following the program (95 °C—1 min; 10 cycles: 95 °C—15 s, 65 °C—30 s, 68 °C—6 min; final elongation at 72 °C—10 min). Alternatively, the second stand was synthesized following the nick translation method. For that, a mix containing 2 μL of RNAse H (NEB, #M0297S), 1 μL of *Escherichia coli* DNA ligase (NEB, #M0205 L), 5 μL of *E. coli* DNA Polymerase (NEB, #M0209 L), 1 μL of dNTP (0 .2mM), 10 μL of 5× Second Stand Buffer (100 mM Tris-HCl (pH 6.9) (AppliChem, #A3452); 25 mM MgCl2 (Sigma, #M2670); 450 mM KCl (AppliChem, #A2939); 0.8 mM β-NAD; 60 mM (NH4)2SO4 (Fisher Scientific Acros, #AC20587); and 11 μL of water was added to 20 μL of ExoI-treated first-strand reaction on ice. The reaction was incubated at 16 °C for 2.5 h or overnight. Full-length double-stranded cDNA was purified with 30 μL (0.6×) of AMPure XP magnetic beads (Beckman Coulter, #A63881) and eluted in 20 μL of water.

### Library preparation and sequencing

The sequencing libraries were prepared by tagmentation of 1–50 ng of full-length double-stranded cDNA. Tagmentation was done either with Illumina Nextera XT kit (Illumina, #FC-131-1024) following the manufacturer’s recommendations or with in-house produced Tn5 preloaded with dual (Tn5-A/B) or same adapters (Tn5-B/B) under the following conditions: 1 μL (11 μM) Tn5, 4 μL of 5× TAPS buffer (50 mM TAPS (Sigma, #T5130), and 25 mM MgCl2 (Sigma, #M2670)) in 20 μL total volume. The reaction was incubated 10 min at 55 °C followed by purification with DNA Clean & Concentrator-5 kit (Zymo Research) and elution in 21 μL of water. After that, tagmented library (20 μL) was PCR amplified using 25 μL NEBNext High-Fidelity 2X PCR Master Mix (NEB, #M0541 L), 2.5 μL of P5_BRB primer (5 μM, Microsynth), and 2.5 μL of oligo bearing Illumina index (Idx7N5 5 μM, IDT) using the following program: incubation 72 °C—3 min, denaturation 98 °C—30 s; 10 cycles: 98 °C—10 s, 63 °C—30 s, 72 °C—30 s; final elongation at 72 °C—5 min. The fragments ranging 200–1000 bp were size-selected using AMPure beads (Beckman Coulter, #A63881) (first round 0.5× beads, second 0.7×). The libraries were profiled with High Sensitivity NGS Fragment Analysis Kit (Advanced Analytical, #DNF-474) and measured with Qubit dsDNA HS Assay Kit (Invitrogen, #Q32851) prior to pooling and sequencing using the Illumina NextSeq 500 platform using a custom ReadOne primer (IDT) and the High Output v2 kit (75 cycles) (Illumina, #FC-404-2005). The library loading concentration was 2.2 pM. The read1 sequencing was performed for 6–21 cycles and read2 for 54–70 cycles depending on the experiment.

### RNA library preparation with TruSeq

TruSeq libraries were prepared with 1 μg of total RNA using the TruSeq Stranded mRNA Library Prep Kit (Illumina, #RS-122-2101) and following the manufacturer’s instructions. Four libraries were paired-end sequenced (75 nt each) with the NextSeq 500 using the Mid Output v2 kit (150 cycles) (Illumina, #FC-404-2001).

### Pre-processing of the data—demultiplexing and alignment

The sequencing reads from our own experiments and public datasets were aligned to the Ensembl r87 gene annotation of the hg38 genome using STAR (RRID:SCR_015899) (version 2.5.3a) [35], and count matrices were generated with HTSeq (RRID:SCR_005514) (version 0.9.1) [36].

The raw reads from BRB-seq experiments carry two barcodes, corresponding to the late and early step multiplexing. The late step multiplexing using Illumina indexes is common to standard protocols and used to separate the libraries. The early barcode is specific to the BRB-seq protocol and is used to separate the multiplexed samples from the bulk data. The first demultiplexing step was performed by the sequencing facility using bcl2fastq software. Then, the data consists of two FASTQ files (R1 and R2). The R2 FASTQ file was aligned to the Ensembl r87 gene annotation of the hg38 genome using STAR with default parameters prior to the second demultiplexing step. Then, using the BRB-seqTools suite (available at http://github.com/DeplanckeLab/BRB-seqTools), we performed simultaneously the second demultiplexing and the count of reads/transcripts (UMI) per gene from the R1 FASTQ and the aligned R2 BAM files. This generated two count matrices (reads and UMI) that were used for further analyses. In parallel, we also used the BRB-seqTools suite for demultiplexing the R1/R2 FASTQ files and producing one FASTQ file per sample. This was required for being able to generate the downsampling of every sample. In this case, FASTQ files were aligned using STAR and HTSeq was used for producing the count matrices.

### mRNA-seq computational analysis and detection of DE genes

All downstream analyses were performed using R (version 3.3.1, https://cran.r-project.org/). Library normalization and expression differences between samples were quantified using the DESeq2 package [36], with cutoff of |FC| ≥ 2 and FDR ≤ 0.05. Further functional enrichments were performed using Fisher’s exact test on Gene Ontology (RRID:SCR_002811) [37], KEGG (RRID:SCR_012773) [38], and Gene Atlas (RRID:SCR_008089) (http://www.genatlas.org/) databases.

### Downsampling of TruSeq and BRB-seq samples

For an unbiased comparison, all samples were randomly downsampled to 1M reads (or as indicated for individual cases). To avoid transferring alignment-related issues to the downstream analyses, we did not downsample at the level of the FASTQ files. Indeed, to be able to keep some information about the reads before their mapping to genes (such as duplicates or UMI), we chose to perform the downsampling at the level of the BAM files, just before performing the htseq-count step. For reproducibility and robustness of the results, we chose to generate 10 downsampled BAM for each replicate.

### TruSeq and BRB-seq comparison

Coverage over the gene body was computed using the RSeQC suite v.2.6.1 (RRID:SCR_005275) [39] with the geneBody_coverage.py script. We used the full list of genes from the hg38 assembly provided on the software web page. ROC and PR AUC plots were produced using the set of 4566 DE genes identified using full paired-end TruSeq samples with the DESeq2 package. This set represents a self-assigned “gold standard,” i.e., the positive set, while the negative set constitutes of all genes expressed as detected by TruSeq but not identified as DE. Then, for every comparison, we applied DESeq2 and used the full list of ranked *p* values to compare to the “gold standard.” False positive rate, true positive rate, and precision (for PR and ROC AUC) were computed for every *p* value cutoff of the ranked *p* value list, thus generating the curves. AUC values were computed using the *rollmean* function of the *zoo* package in R. Mitochondrial RNA content (called MT-rRNA content in the figures) was assessed using only two MT-rRNA genes that are known to be the main representatives of any mitochondrial contamination: *MT-RNR1* and *MT-RNR2*.

### Power simulation analysis

The power simulation was conducted using the powsimR R package [26]. We used control hASC samples (2 T0) from TruSeq and BRB-seq, control (3 DMSO) SCRB-seq samples from the LCL dataset, and 6 SCRB-seq control samples from Hafner et al. [16, 40] (SRR3384233, SRR3384235, SRR3384197, SRR3384205, SRR3384238, SRR3384227), Cacchiarelli et al. [15, 41] (SRR2044011, SRR2044039, SRR2044038, SRR2044023, SRR2044034, SRR2044037), Kilens et al. [18, 42] (A3H04LEP09_L8A1, A4C08LEP20_L8A1, A4C06LEP09_L8A1, A4C07LEP15_L8A1, A6F05LEP15_L8A1, A3G01LEP11_L8A1), and Xiong et al. [14, 43] (CTRL_Rep_1, CTRL_Rep_2, CTRL_Rep_3, CTRL_Rep_4, CTRL_Rep_5, CTRL_Rep_6). Then, we randomly downsampled all replicates to 1M reads, twice for the LCL dataset, thrice for the hASC dataset, and once for the four published datasets, thus resulting into six 1M reads replicates for every study. Then, we created the simulation model by estimating empirically the mean dispersion and dropout relationships using the powsimR package with the “bulk RNA-seq,” “NB” (inferred negative binomial distribution), and “MR” (DESeq2) normalization parameters. Using this model, we then simulated expression data of 10,000 genes for *n* replicates (*n* in [5, 19, 44]). Amongst the 10,000 genes, we simulated 10% differentially expressed genes with log fold change drawn from a narrow gamma distribution. For every study, we simulated 100 random datasets that were then tested for differential expression using DESeq2 at FDR 5% threshold, from which the average true positive rate (TPR) was calculated. For reproducibility purpose, the R script used for this step is available as Additional file 4.

## Additional files

Additional file 1: **Figure S1.** Evaluation of data produced with SCRB-seq. **Figure S2.** Experimental design and performance assessment of the method. **Figure S3.** Comparisons of different BRB-seq workflows with variable RT enzymes and second-strand generation methods. **Figure S4.** Assessment of BRB-seq performance relative to TruSeq. **Figure S5.** The RNA fragment size profiles of intact samples and their degraded counterparts after one or two minutes of fragmentation. (PDF 1285 kb)

Additional file 2: **Table S1.** List of publications referencing the Soumillon et al. 2014 study in the context of bulk RNA profiling using the SCRB-seq protocol. **Table S2.** List of LCL samples from the GBR population in the 1000 Genome Project. **Table S3.** The detailed cost structure of the BRB-seq protocol. **Table S4.** List of samples used in the manuscript. **Table S5.** List of primers used RT-qPCR. **Table S6.** BRB-seq barcoded oligo-dT primers (BU3). **Table S7.** Primers used for BRB-seq library preparation. (XLSX 34 kb)

Additional file 3: Analysis of raw sequencing data using BRB-seq Tools. (PDF 110 kb)

Additional file 4: R script used to generate the simulated dataset with powsimR package [28]. (R 6 kb)

## Abbreviations

DE: Differentially expressed
DS: Double stranded
dUTP: 2′-Deoxyuridine, 5′-triphosphate
hASCs: Human adipose stromal cells
n.s.: Non-significant
PR AUC: Precision recall area under curve
qPCR: Quantitative polymerase chain reaction
ROC AUC: Receiver operating characteristic area under curve
RT: Reverse transcription
TSO: Template switch oligo
UMI: Unique molecular identifier
