# Supplementary material for: BRB-seq: ultra-affordable high-throughput transcriptomics enabled by bulk RNA barcoding and sequencing
Source: Genome Biol. 2019 Apr 19;20:71. doi: 10.1186/s13059-019-1671-x (PMC6474054; doi:10.1186/s13059-019-1671-x)
Supplement: Supplementary file 1 — Figure S1. Evaluation of data produced with SCRB-seq. Figure S2. Experimental design and performance assessment of the method. Figure S3. Comparisons of different BRB-seq workflows with variable RT enzymes and second-strand generation methods. Figure S4. Assessment of BRB-seq performance relative to TruSeq. Figure S5. The RNA fragment size profiles of intact samples and their degraded counterparts after one or two minutes of fragmentation. (PDF 1285 kb) [file 13059_2019_1671_MOESM1_ESM.pdf]

Additional File 1: Supplementary figures

BRB-seq: ultra-affordable high-throughput transcriptomics enabled by bulk RNA barcoding and sequencing

Daniel Alpern*1,2, Vincent Gardeux*1,2, Julie Russeil1, Bastien Mangeat3, Antonio C. A. Meireles-Filho1,2, Romane Breysse1, David Hacker4 and Bart Deplancke1,2,#

1 Institute of Bioengineering, School of Life Sciences, École Polytechnique Fédérale de Lausanne (EPFL), CH-1015 Lausanne, Switzerland

2 Swiss Institute of Bioinformatics, CH-1015 Lausanne, Switzerland

3 Gene Expression Core Facility, School of Life Sciences, École Polytechnique Fédérale de Lausanne (EPFL), CH-1015 Lausanne, Switzerland

4 Protein Expression Core Facility, School of Life Sciences, École Polytechnique Fédérale de Lausanne (EPFL), CH-1015 Lausanne, Switzerland

* Shared first author

# To whom correspondence should be addressed: bart.deplancke@epfl.ch

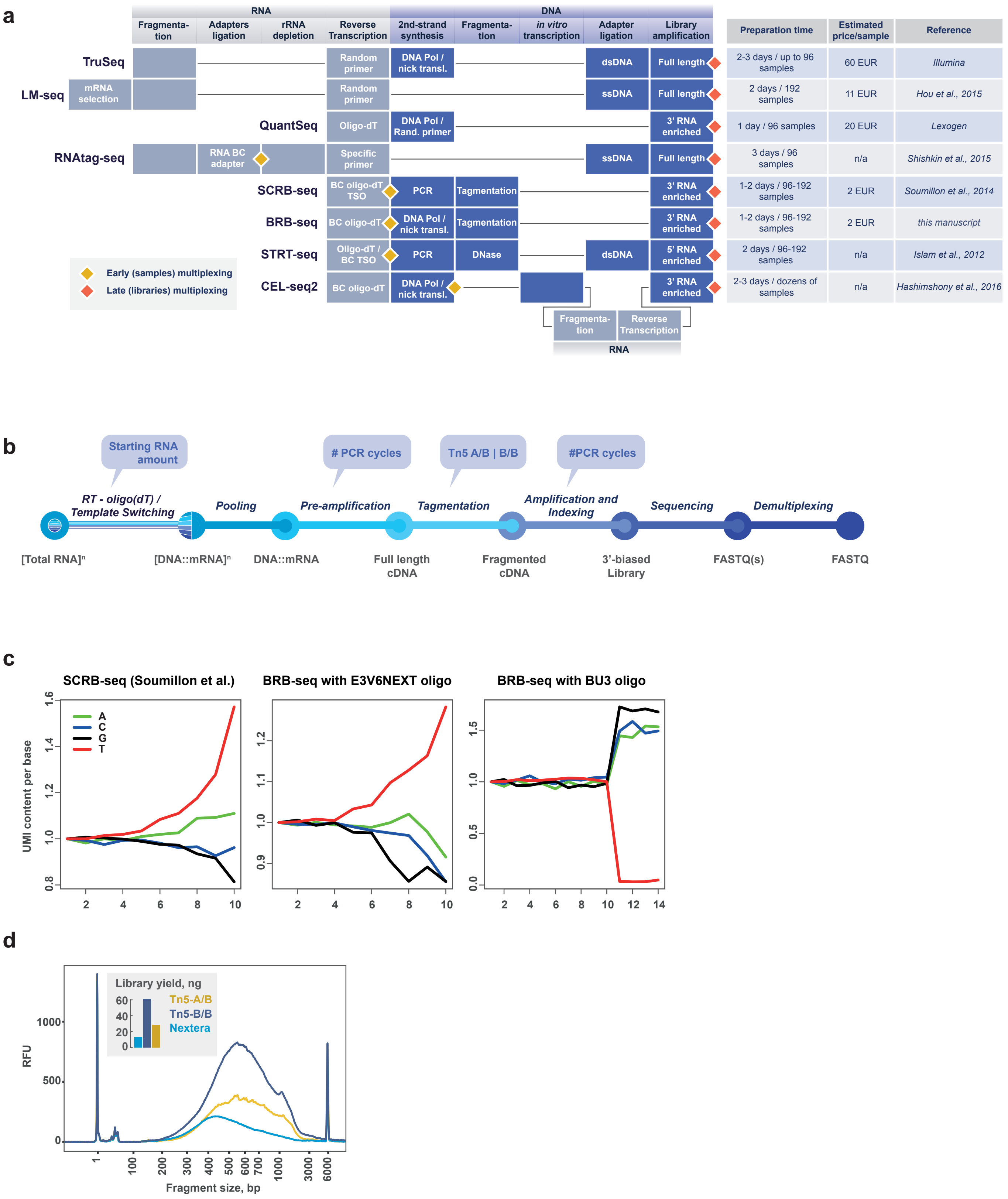

Figure S1. Evaluation of data produced with SCRB-seq. a. State-of-the-art bulk and single-cell RNA-seq approaches allowing different levels of sample multiplexing. Every box corresponds to a standardized step of the protocol. Diamonds represent the multiplexing steps: early (yellow) or late (red). The right table shows the throughput of each protocol and the estimated library preparation cost per sample in Euros. Legend: BC, barcoded; TSO, template switch oligo; ssDNA: single-stranded DNA; dsDNA: double-stranded DNA. **b.** The key steps of the initial SCRB-seq protocol, with four of these considered critical for optimization: i) the amount of input RNA, ii) the number of pre-amplification cycles post RT, iii) the Tn5 enzyme type, and iv) the number of amplification cycles post tagmentation. **c.** The UMI base content in i) SCRB-seq samples (here D1T0A sample) with E3V6NEXT

10bp UMI oligo ii) BRB-seq performed with bulk RNA and E3V6NEXT oligo, and iii) BRB-seq with our modified BU3 oligo, bearing a 15bp UMI, the five last nucleotides being non-T. **d.** The library profiles and yields after tagmentation with various Tn5 enzymes: in-house made Tn5-A/B and Tn5-B/B and Illumina Nextera.

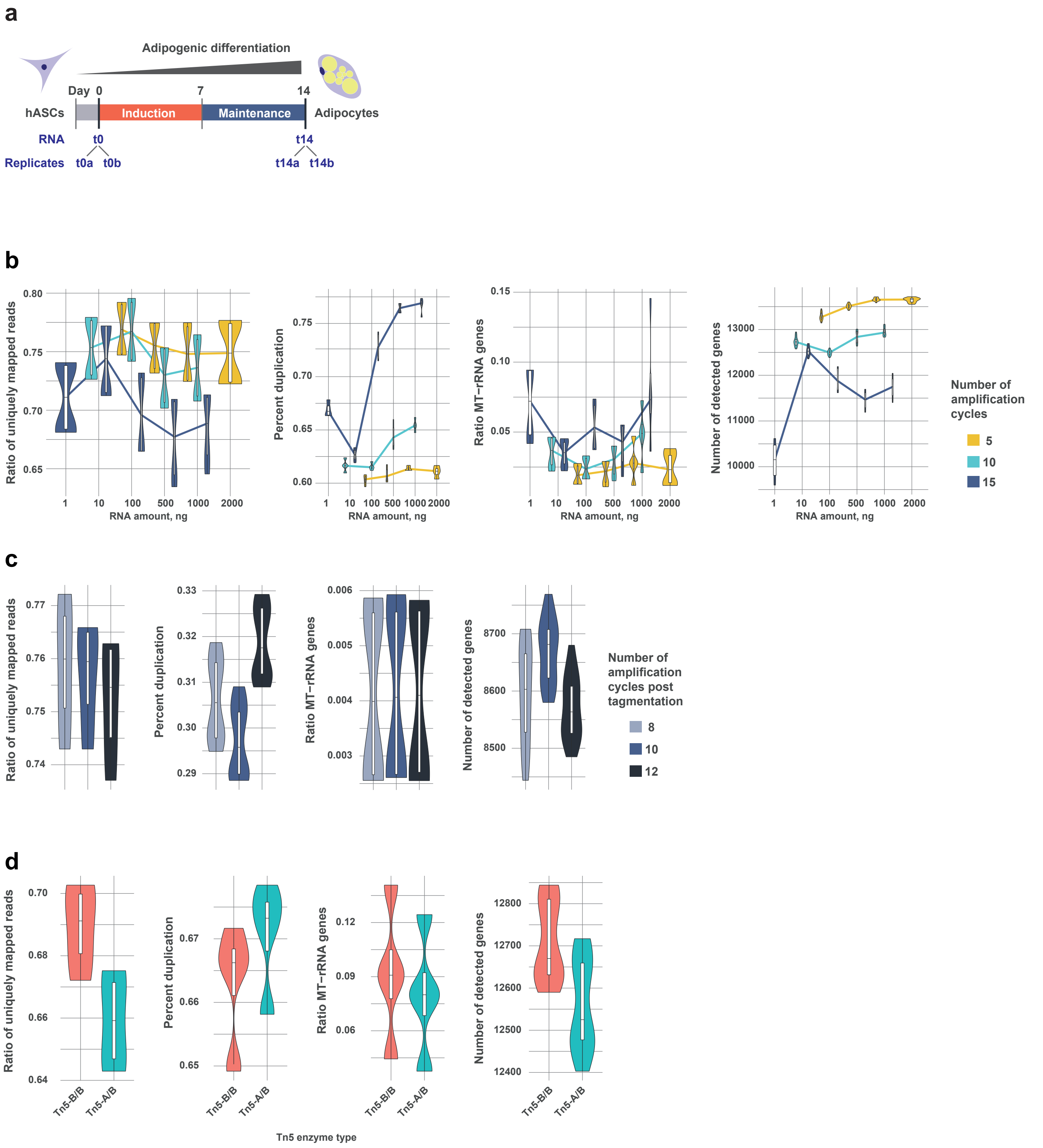

Figure S2. Experimental design and performance assessment of the method. a. The experimental design for preparation of the RNA samples. Human adipocyte stromal cells (hASCs) were differentiated using the adipogenic induction and maintenance cocktails during 7 days each to obtain adipocytes. The cells at both differentiation time points (t0 and t14) were used for RNA extraction. To prepare the libraries, two aliquots of each of two RNA samples at different dilutions (1 – 2000 ng) were used as technical replicates. **b.** Assessment of the impact of the initial RNA amount and number of pre-amplification cycles. **c.** Assessment of the impact of the number of amplification cycles for the second amplification (post tagmentation). **d.** Assessment of the impact of Tn5 enzyme type. For the **b. c. & d.** performances were evaluated using different quality measures: uniquely mapped reads, level of duplication, rate of MT-rRNA reads, and complexity (number of detected genes). For an unbiased comparison, libraries were downsampled to one million (b.) or 100k (c. and d.) single-end reads (see Methods)

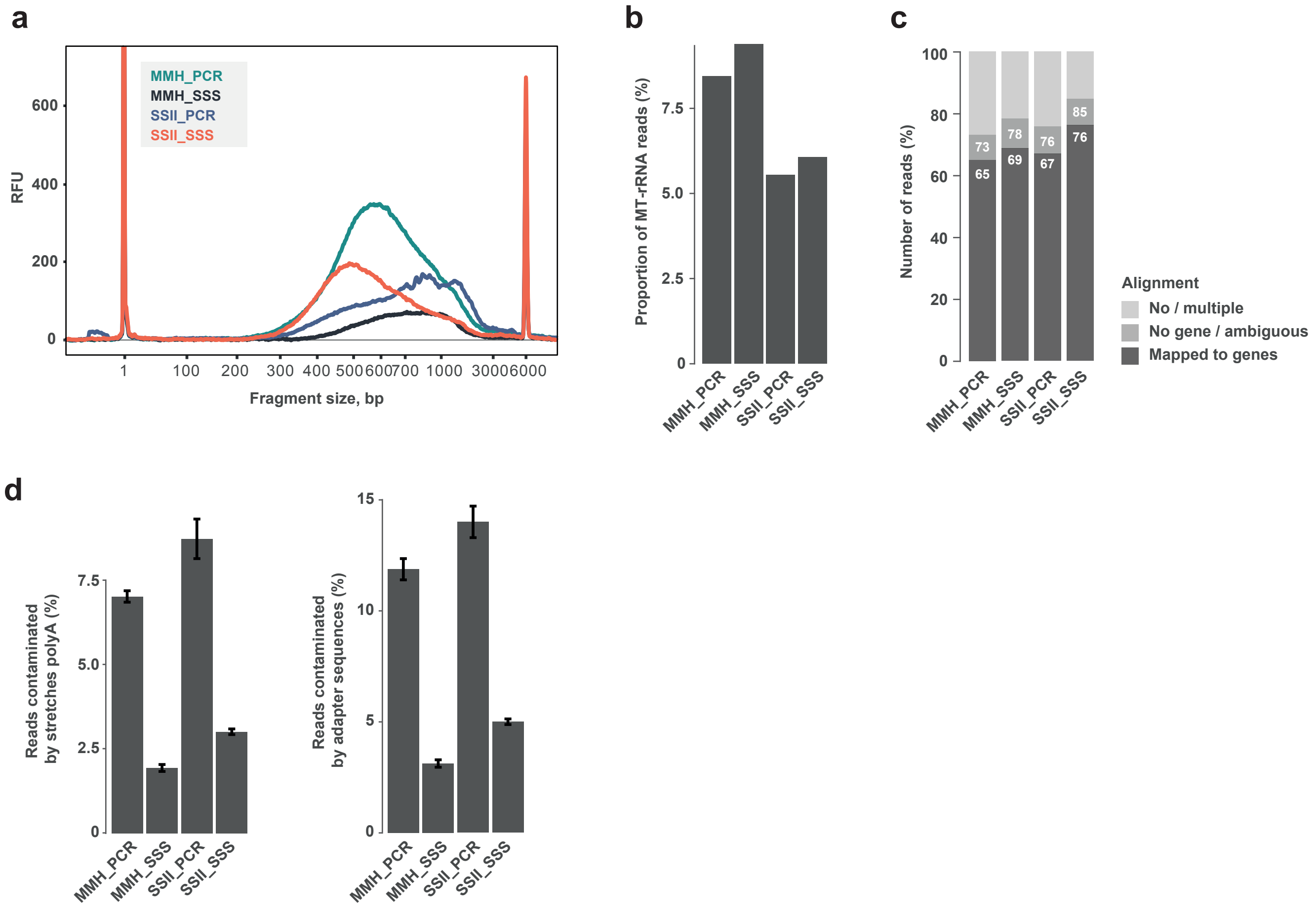

Figure S3. Comparisons of different BRB-seq workflows with variable RT enzymes and second strand generation methods. a. Comparison of fragment size profiles. **b.** Comparison of the ratio of reads assigned to MT-rRNA transcripts. **c.** Comparison of read alignment performances. The no/multiple alignment values are derived from the STAR alignment, and no gene/ambiguous and mapped to genes correspond to the annotation of the reads to the genes by Htseq. **d.** Comparison of the ratio of reads that are found contaminated whether by BRB-seq adapter sequences or stretches of more than 6 polyAs during the trimming step (performed using the BRB-seqTools software). *Legend: MMH, Maxima Fermentas Minus H Enzyme; SSII, Superscript II Enzyme; SSS: Second-Strand Synthesis using Nick translation; PCR: pre-amplification by Polymerase Chain Reaction.*

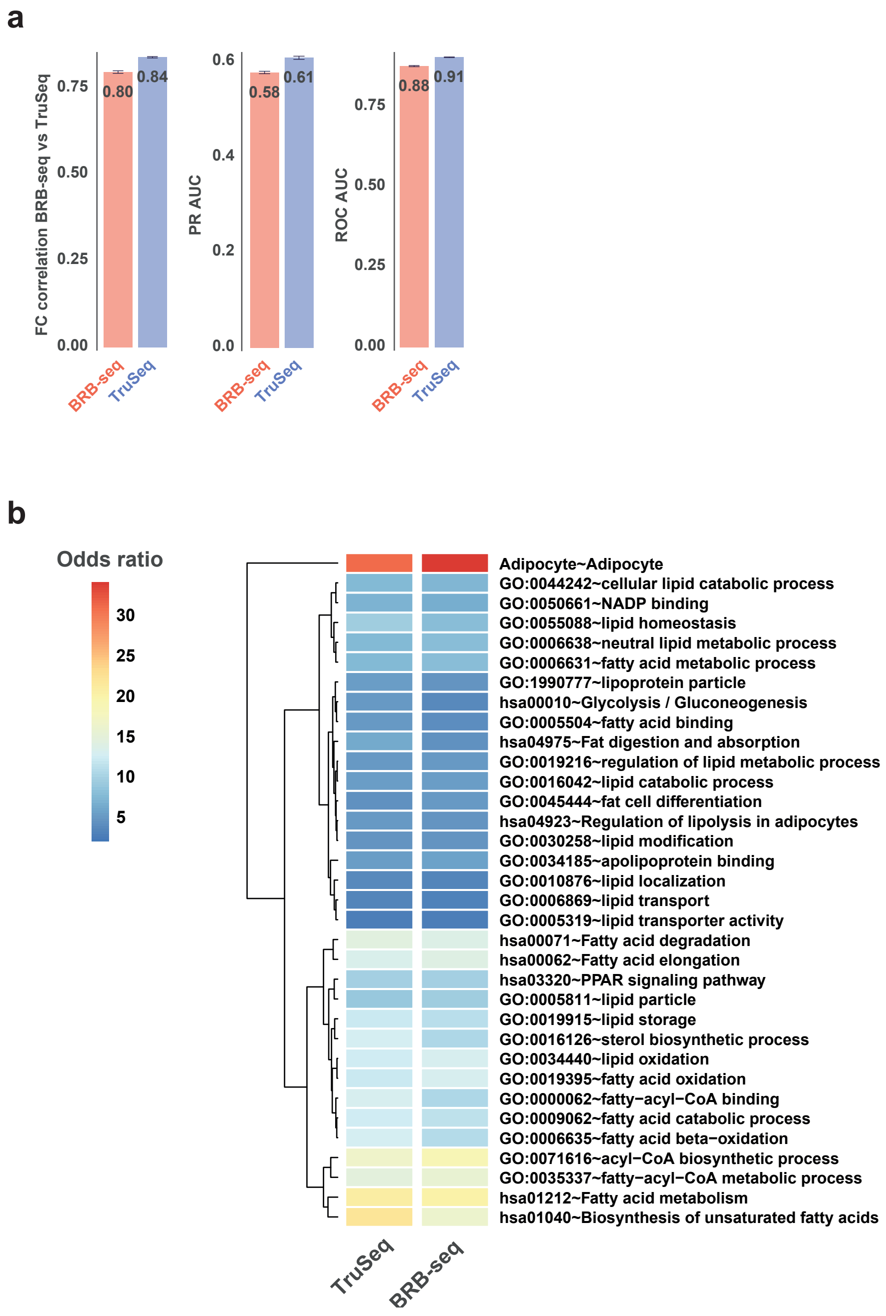

Figure S4. Assessment of BRB-seq performance relative to TruSeq. a. Evaluation of BRB-seq's performance relative to TruSeq represented by different quality markers and using the data down-sampled to 1M single-end reads. Both samples are compared to the "gold standard" TruSeq ~30M paired-end reads. **b.** Functional enrichment of the identified differentially expressed genes (FC > 2 & FDR < 5%) between TruSeq and BRB-seq. Functional Enrichment was performed on gene sets linked to adipocyte differentiation in different databases: Gene Ontology (Biological Processes, Molecular Functions and Cellular Components), and KEGG pathways. Odds ratio is calculated using a Fisher's Exact Test.

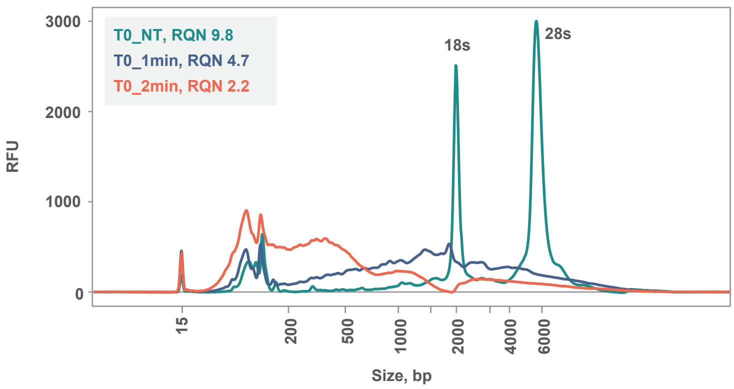

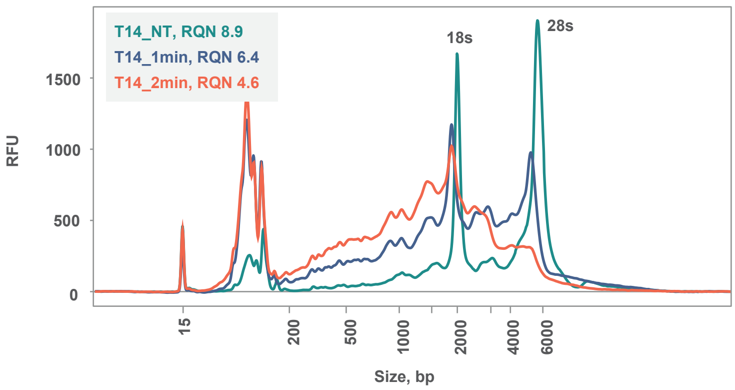

Figure S5. The RNA fragment size profiles of intact samples and their degraded counterparts after one or two minutes of fragmentation. Left panel represents the t0 samples (pre-adipocytes), while the right panel features the t14 samples (differentiated adipocytes). *Legend: RQN, RNA quality number (maximum is 10).*
