# Supplementary material for: BRB-seq: ultra-affordable high-throughput transcriptomics enabled by bulk RNA barcoding and sequencing
Source: Genome Biol. 2019 Apr 19;20:71. doi: 10.1186/s13059-019-1671-x (PMC6474054; doi:10.1186/s13059-019-1671-x)
Supplement: Supplementary file 3 — Analysis of raw sequencing data using BRB-seq Tools. (PDF 110 kb) [file 13059_2019_1671_MOESM3_ESM.pdf]

Additional File 3: BRBseq Tools

BRB-seq: ultra-affordable high-throughput transcriptomics enabled by bulk RNA barcoding and sequencing

Daniel Alpern*1,2, Vincent Gardeux*1,2, Julie Russeil1, Bastien Mangeat3, Antonio C. A. Meireles-Filho1,2, Romane Breysse1, David Hacker4 and Bart Deplancke1,2,#

1 Institute of Bioengineering, School of Life Sciences, École Polytechnique Fédérale de Lausanne (EPFL), CH-1015 Lausanne, Switzerland

2 Swiss Institute of Bioinformatics, CH-1015 Lausanne, Switzerland

3 Gene Expression Core Facility, School of Life Sciences, École Polytechnique Fédérale de Lausanne (EPFL), CH-1015 Lausanne, Switzerland

4 Protein Expression Core Facility, School of Life Sciences, École Polytechnique Fédérale de Lausanne (EPFL), CH-1015 Lausanne, Switzerland

* Shared first author

# To whom correspondence should be addressed: bart.deplancke@epfl.ch

Analysis of raw sequencing data using BRB-seq Tools

BRB-seq Tools is command-line suite for the pre-processing of BRB-seq data (bulk RNA-seq). Provided as a single executable Java file it enables to generate a gene count matrix table from FASTQ files produced by Illumina sequencer. BRB-seq Tools is downloadable as a .JAR file that contains all required materials and can be run on any terminal.

Download BRB-seq Tools

The BRB-seq Tools can be downloaded from GitHub following this link.

Sequencing output

The sequencing of a BRB-seq library produces two FASTQ files:

- R1 FASTQ file: This file contains the barcodes and UMIs of the BRBseq construct. In the default primer design the barcode comes before the UMI sequence. The sequencing of UMIs is optional and depends on the project. Therefore, R1 FASTQ file contain the reads of length 6 (if only barcodes is sequenced) or up to 21 (in both barcode [6nt] and UMI [15nt] is sequenced).
- R2 FASTQ file: This file contains the actual cDNA fragment sequencing reads. It should carry the exact same number of reads (and read names) as the R1 file.

Necessary software and resources

BRB-seq Tools is a suite dedicated for the generation of the output gene count/UMI matrix. Any additional information about this software can be found at the GitHub page.

To apply BRB-seq Tools, the sequencing reads should be aligned to the reference genome. We recommend using the STAR aligner, but any other alignment method can be applied.

For further analyses (filtering, normalization, dimension reduction, clustering, differential expression), we recommend using ASAP web portal that you can freely access at asap.epfl.ch.

Although the suite can be run on a desktop computer with installed Java (version 1.8 or higher) using the terminal (Linux, iOS, PC), the alignment step and initial genome index generation by STAR aligner may require up to 32Gb of RAM (for large genomes such as human).

Step by step protocol (using STAR)

A. Generation of genome index

The index of the reference genome is generated once for a given genome assembly. Therefore, the step **A** can be skipped if you already possess an indexed genome.

1. Download last release of your species of interest .FASTA file from Ensembl or any other preferred database (here we used *Homo sapiens* from Ensembl).

```
wget ftp://ftp.ensembl.org/pub/release-
90/fasta/homo_sapiens/dna/Homo_sapiens.GRCh38.dna.primary_assembly.fa.gz
```

2. Unzip the file

```
gzip -d Homo_sapiens.GRCh38.dna.primary_assembly.fa.gz
```

3. Download last release of Homo sapiens .GTF file from Ensembl

```
wget ftp://ftp.ensembl.org/pub/release-
90/gtf/homo_sapiens/Homo_sapiens.GRCh38.90.gtf.gz
```

4. Unzip the file

```
gzip -d Homo_sapiens.GRCh38.90.gtf.gz
```

5. Create a folder STAR_Index

```
mkdir STAR_Index/
```

6. Generate the STAR genome index (in 'STAR_Index' folder). NOTE: This requires up to ~30G RAM depending on the genome used.

```
STAR --runMode genomeGenerate --genomeDir STAR_Index/ --genomeFastaFiles
Homo_sapiens.GRCh38.dna.primary_assembly.fa --sjdbGTFfile
Homo_sapiens.GRCh38.90.gtf
```

<u>NOTE:</u>

- You can add the argument '--runThreadN 4' for running 4 threads in parallel (or more if your computer has enough cores).
- If the genome is not human or mouse, you may need to add/tune the argument '--genomeSAindexNbases xx' with xx = log2(nbBasesInGenome)/2 – 1.
  (*For e.g. for Drosophila melanogaster xx ~ 12.43, thus you should add the argument '--genomeSAindexNbases 12')*

B. Running BRB-seq Tools

The BRB-seq Tools can be used to trim the sequencing reads to remove the adapter or polyA reads (optional step 1). The suite employs the aligned bulk R2 BAM file, produced by an aligner (STAR), and corresponding R1 FATSQ, containing barcodes/UMI information, to create a count matrix with the number of reads per gene information for every sample. The user should provide a tab delimited text file ('lib_example_barcodes.txt') with two columns and a header (Name, B1) with the list of sample names and corresponding barcode sequences. See an example below or download this file.

```
Name            B1
G1_a            AAACAT
G1_b            AAATCA
G1_c            AACATA
G1_d            AAGTTA
...             ...
```

1. [Optional] Trim the read containing the sequence fragments (generates a 'lib_example_R2.trimmed.fastq.gz' file).

```
java -jar BRBseqTools.jar Trim -f lib_example_R2.fastq.gz
```

2. Create the output folder

```
mkdir BAM/
```

3. Use the aligner (e.g. STAR, with the genome index file supplied) to map the sequencing reads to the genome. Align only the R2 FASTQ file (using STAR, no sorting/indexing is needed)

```
STAR --runMode alignReads --genomeDir STAR_Index/ --outFilterMultimapNmax 1 --
readFilesCommand zcat --outSAMtype BAM Unsorted --outFileNamePrefix BAM/ --
readFilesIn lib_example_R2.trimmed.fastq.gz
```

NOTE:

- You can add the argument '--runThreadN 4' for running 4 threads in parallel (or more if your computer has enough cores).
- The '--outFilterMultimapNmax 1' option is recommended for removing multiple mapping reads from the output BAM

4. Rename the output aligned BAM

```
mv BAM/Aligned.out.bam BAM/lib_example_R2.bam
```

5. Demultiplex and generate output count/UMI matrix

```
java -jar BRBseqTools.jar CreateDGEMatrix -f lib_example_R1.fastq.gz -b
BAM/lib_example_R2.bam -c lib_example_barcodes.txt -gtf
Homo_sapiens.GRCh38.90.gtf -p BU -UMI 14
```

NOTE:

- The '**-p**' parameter specifies the pattern in the R1 FASTQ file. If only 6nt **B**arcode was sequenced, then you should use '**-p B**'. In case 10nt **U**MI was sequenced along with the **B**arcode, use '**-p BU**' followed by the '**-UMI 10**' parameter to specify the length of the UMI.
- In any case, the length of the pattern (B or BU) should match the total length of the sequence read in R1. Therefore, the pattern can be extended as needed by using a certain number of '?' character. For e.g., you sequenced 6nt barcode, followed by 10nt UMI but the R1 contains extra 5nt after the UMI, which makes the length of the sequence in R1 being 21nt. In this case, you should use '**-p BU????? -UMI 10**' to specify that after the UMI, there are 5nt (?????) that will not be used for the analysis.
- 'lib_example_barcodes.txt' is a tab delimitated text file with two columns and a header (Name, B1) which should be created by the user and contain the barcode sequence and the corresponding sample name. See an example below or you can download this file.

At the end of the process the script generates 'output.dge.reads.txt' count matrix file or 'output.dge.umis.txt' if UMIs ('-p BU') were included in the analysis. Every line of the matrix corresponds to a gene, and every column to a sample. Each cell is thus the corresponding gene expression in a given sample. You can also load the count/UMI matrices in R to perform further analyses. Or you can upload this file to https://asap.epfl.ch and run the analysis pipeline online.
